# The temporal geographically-explicit network of public transport in Changchun City, Northeast China

**DOI:** 10.1038/sdata.2019.26

**Published:** 2019-02-26

**Authors:** Qiuyang Huang, Yongjian Yang, Zhilu Yuan, Hongfei Jia, Liping Huang, Zhanwei Du

**Affiliations:** 1 College of Computer Science and Technology, Jilin University, Changchun, 130012, China; 2 Research Institute for Smart Cities, School of Architecture and Urban Planning, Shenzhen University, Shenzhen, China; 3 College of Transportation, Jilin University, Changchun, 130012, China

**Keywords:** Geography, Information technology, Complex networks

## Abstract

The vehicle trajectory data is a feasible way for us to understand and reveal urban traffic conditions and human mobility. Therefore, it is extremely valuable to have a fine-grained picture of large-scale vehicle trajectory data, particularly in two different modes, taxis and buses, over the same period at an urban scale. This paper integrates the trajectory data of approximately 7,000 taxis and 1,500 buses in Changchun City, China and accesses the temporal geographically-explicit network of public transport via sequential snapshots of vehicle trajectory data every 30 seconds of the first week in March 2018. In order to reveal urban traffic conditions and human mobility, we construct two-layer urban traffic network (UTN) between these two different transport modes, take crossings as nodes and roads as edges weighted by the volume or average speed of vehicles in each hour. We released this temporal geographically-explicit network of public transport and the dynamics, weighted and directed UTN in simple formats for easy access.

## Background & Summary

Urban public transport plays an important role in citizens’ daily life as the infrastructure of social economics. With the development and application of big data technology in the field of intelligent transport systems^1,2^ and urban computing^3,4^, the data-driven modelling method has attracted many researching interests, such as human mobility^5^ and complex dynamical systems^6^. Under the background of the data driven model, the vehicle trajectory data can provide a new understanding of the city^7–11^, which consists of the location information together with the positioning time stamp generated by the equipped GPS devices. Trajectory data can be processed as the materials for effective methods to issue the urban problems, such as travel demand analysis^12,13^, discovery of community detection^14,15^, analysis of human movements^16,17^, especially, it plays an important role to solve the problem of urban traffic congestion^7,18–20^.

There are many kinds of public transport modes in the city, such as buses, taxis, subway, light rail and so on. Among them, the trajectory data of taxis and buses can reflect the operation of urban. But in many cities, these different urban traffic modes are often managed by a number of companies alone, there is a lack of unified analysis and research on various modes of transportation, only a few studies focus on multilayer aspects^21–24^ of the public transport.

In this paper, we integrate the fine-grained trajectory data of taxis and buses from Changchun municipal Engineering Design & Research Institute. The dataset contains approximately 7,000 taxis and 1,500 buses in Changchun city, with updated locations every 30 seconds during a one-week period from 5 March 2018.

Each vehicle’s movement trace contains the time series of locations, which the vehicle passes through on the road network. During the collection of vehicle trajectory data, there are some missing information due to equipment failure, system failure and poor signal, such as data error and duplication, data drift and data missing.

Informed by the corrected the data (the details are in the Methods section), we use the map-matching algorithm^25,26^ to match the trajectory points available in the dataset to the road network and estimate the hourly traffic volume of each road as well as the average speed. After that, we construct a two-layer urban traffic network (UTN) by taking crossings as nodes and roads as edges weighted by the volume or average speed of vehicles in each hour.

To ensure the access to the open data, we save the two-layer UTN information of buses and taxis in the .txt files separated by commas. For each traffic mode has 2 files, each one records UTN of buses and taxis with rows of road ID, and the weight of 24 hours, one is the number of vehicles passing through the road, and the other one is the average passing speed of the road. In addition, we provide the ArcGIS shapefiles for the road segments, bus stations and the districts of Changchun city in our dataset.

## Methods

### Original data sources

One-week vehicle trajectory data of two modes, taxis and buses, are provided by Changchun municipal Engineering Design & Research Institute in Changchun city since 5 March 2018. All these taxis and buses are equipped with GPS devices, which can upload their location information to the designated server every 30 seconds. Each record in the raw files contains the information of vehicle ID, longitude, latitude and timestamp. For each vehicle, all of its records sort by time will constitute the whole trajectory of the vehicle in one day. In order to manage data and build UTN conveniently, the data are sorted by time and split into a separate file every 5 minutes to compose 288 files in a single day. It contains approximately 7,000 taxis and 1,500 buses in original data sources.

We construct two-layer UTN through these original data sources by taking crossings as nodes and roads as edges whose weights denote the volume or average speed of vehicles in each hour. Besides, we also provide ArcGIS shapefiles for the road network, bus stations and administrative districts of Changchun city. The institute kindly accepted to grant us the rights of sharing these anonymized data, and we release the two-layer UTN dataset in the figshare websites as Open Data under Attribution 4.0 International (CC BY 4.0) licence.

### Data correction

Because of equipment failures, system failures, bad signals and so on, there are some problems with the original data as follows:

- **Data error and duplication:** In original data, some vehicles ID were replaced with ‘0’ or ‘ffffff’, and the latitude and longitude fields of some records are empty. Besides, some of the records are repeated. These data are deleted at first.
- **Data drift:** Data drift denotes the scenarios when the record’s location is far away from the actual location, perhaps due to the poor signal or GPS positioning error. For each vehicle, we sort its trajectory data by time. We delete any data point if it is far away from more than two of its four neighbouring points (two early and two later) than a certain threshold *d*_*θ*_. The threshold is estimated by $d_{\theta \;}=v_{\theta }*\sqrt{{\left(t-{t\prime }^{}\right)}^{2}}$, where *t* and ${t\prime }^{}$ represents the timestamp of two points respectively. *v*_*θ*_ represents the maximum speed in each layer (120 km/h in bus-layer and 180 km/h in taxi-layer).
- **Data missing:** Because of the system failure and the existence of error data, the trajectory data is missing in some time. We define that if the time interval between two continuous points in a trajectory is more than 5 minutes, it is considered to be a lack of data. For taxis, we use the shortest path to connect these two points. The bus line is fixed, so we fill data through the historical trajectory.

### Map-matching

Map-matching is the fundamental process of matching a sequence of trajectory points with the corresponding road on a digital map. After our data correction, the error between most trajectory points and their real locations is very small, so we first match all the GPS points to its nearest road segment. To avoid mismatching caused by positioning error and road intersections, we use a sliding window with a width of 3 to scan each trajectory point. The three points in the sliding window are labelled as pa, pb and pc in sequence, and their matching roads are ra, rb and rc respectively. If rb is different from ra and rc, and ra and ra are the same roads or they are connected, then rb will be deleted.

### Data privacy protection

The original data provided by Changchun municipal Engineering Design & Research Institute has been processed anonymously. On this basis, we construct the two-layer UTN through the original trajectory data which include precise GPS and time information. Finally, we just release the two-layer UTN data which derived from the original trajectory data to protect the privacy of the individuals.

### Defining two-layer UTN

The volume and average speed on the road network reveal the urban traffic condition and human mobility. Accordingly, we take these two indicators as weight respectively to construct two-layer UTN. Some definitions are given below.

Definition 1

(*traffic volume*): The traffic volume of road r is the number of vehicles passing through road r in time t.

Definition 2

(*average speed*): The average speed of road r as the average speed of all vehicles passing through the road r in time t, the unit is kilometre per hour.

Definition 3

(*active vehicle*): We define an active vehicle to be a taxi or bus which moves in a distance of more than 100 meters in 5 minutes.

Definition 4

(*road network*): A road network is defined as a directed graph (*V*, *E*), where *V* is a set of vertices representing the crossings of the road segments, and *E* is a set of edges representing road segments.

We use the map-matching algorithm mentioned above to match the active vehicles’ trajectories to the road network at first. And then, we calculate the traffic volume and average speed of each road segment in one hour. Finally, we take the crossings as the nodes and the road segments as the edges, and use the traffic volume and the average speed as weights respectively, each public traffic mode as a single layer, we finally construct two-layer UTN.

### Code availability

We share our codes for data correction, data analysis and UTN construction in GitHub (Data Citation 1). The detailed description of the codes is in the README.md (Data Citation 1).

## Data Records

This dataset is stored as a single ‘.rar’ file in the figshare websites (Data Citation 2). It describes two-layer UTN in Changchun city of northeast China by using vehicle trajectory data of approximately 7,000 taxis and 1,500 buses. There are 4 files for each day, each one records UTN of buses and taxis with rows of road ID, and the weight of 24 hours. Two kinds of weights are recorded in different files of buses and taxis, one is the number of vehicles passing through the road, and the other one is the average passing speed of the road. In addition, we provide the ArcGIS shapefiles for the road segments, bus stations and the districts of Changchun city.

Finally, we use some folders to group these files together and store them as a single rar file (Data Citation 2). The first folder (Bus-utn) contains 2 subfolders (Bus-utn-volume and Bus-utn-speed), and each subfolder includes 7 files (Day-#-bus-volume.txt and Day-#-bus-speed.txt) of buses in UTN for each day of a week. The second folder (Taxi-utn) also contains 2 subfolders (Taxi-utn-volume and Taxi-utn-speed), and each subfolder includes 7 files (Day-#-taxi-volume.txt and Day-#-taxi-speed.txt) of taxis in UTN for each day of a week. The third folder (Shapefiles) contains 3 folders (roadNetwork, stations and districts) which denote the ArcGIS shapefiles for the road segments, bus stations and the districts of Changchun city.

### Day-#-bus-volume.txt

In bus UTN, each row represents the road’s volume of 24 hours in one day. The format for this file is the following: road ID, v00, v01, …, v23.

- road ID: Road ID in the road network;
- v-##: The weight represents the volume of buses passing through the road in the hour of starting from ##:00 to ##:59 in that day.

### Day-#-bus-speed.txt

In bus UTN, each row represents the road’s average speed of 24 hours in one day. The format for this file is the following: road ID, s00, s01, …, s23.

- road ID: Road ID in the road network;
- v-##: The weight represents the average speed of buses passing through the road in the hour of starting from ##:00 to ##:59 in that day.

### Day-#-taxi-volume.txt

In taxi UTN, each row represents the road’s volume of 24 hours in one day. The format for this file is the following: road ID, v00, v01, …, v23.

- road ID: Road ID in the road network;
- v-##: The weight represents the volume of taxis passing through the road in the hour of starting from ##:00 to ##:59 in that day.

### Day-#-taxi-speed.txt

In taxi UTN, each row represents the road’s average speed of 24 hours in one day. The format for this file is the following: road ID, s00, s01, …, s23.

- road ID: Road ID in the road network;
- v-##: The weight represents the average speed of taxis passing through the road in the hour of starting from ##:00 to ##:59 in that day.

### RoadNetwork

The ArcGIS shapefiles for the road network, it includes two folders of edges and nodes, the nodes represent junctions, while edges represent road segments.

### Nodes:

- FID: Row number;
- lon: The length of the road;
- lat: The ID of two endpoints of the road;
- osmid: Unique identification of nodes

### Edges:

- road_id: Road ID in the road network;
- length: The length of the road;
- from and to: The osmid of two endpoints of the nodes;

### Stations

The ArcGIS shapefiles for the bus stations, each row represents a station in a bus line. It includes the following attributes:

- FID: Each row has an ID;
- id: the station ID;
- line_id: The line ID of the bus station;
- line_name: The name of the line;
- name: The bus station’s name;
- sequence: The ordering of the bus station in the whole line;
- x and y: The longitude and latitude of the bus station.

### Districts

The ArcGIS shapefiles for the districts, which includes the information of population and socioeconomic status of each district in 2014.

- FID: Row ID;
- place_name: The name of each district;
- population: The population of each district in 2014.
- birth_rate and mortality: Birth rate and mortality of each district in 2014;
- GDP and GDP_rate: The GDP and GDP growth rate of each district in 2014;

## Technical Validation

**Figure 1:**
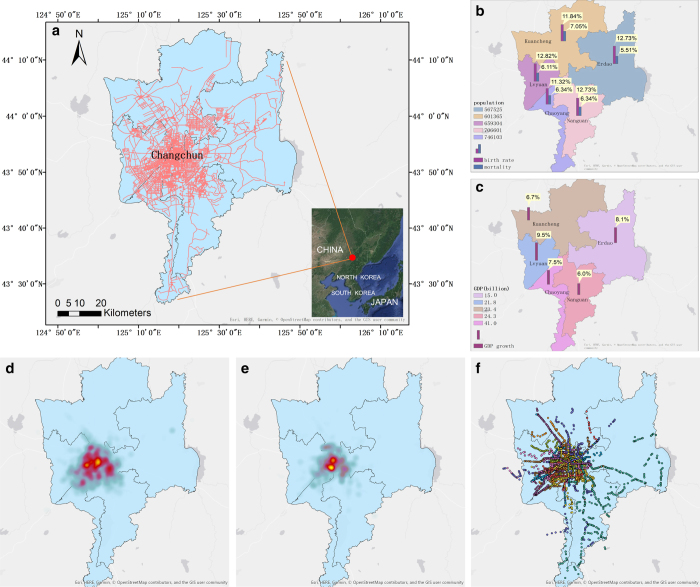
Basic information of Changchun city. (**a**) shows the road network of Changchun city on the map, and the bottom right corner of (**a**) shows the position of Changchun city in China. (**b**) shows population, birth rate and mortality of each district in 2014. (**c**) shows GDP and GDP growth of each district in 2014. (**d**) shows the heat map of 7,000 taxis distribution at 8:00 am on 5 March 2018. (**e**) shows the heat map of 1,500 buses distribution at the same time. (**f**) shows the geographical distribution of bus stations. The spatial map was created using OpenStreetMap online platform (http://www.openstreetmap.org/) (© OpenStreetMap contributors) under the license of CC BY-SA (http://www.openstreetmap.org/copyright). More details of the licence can be found in http://creativecommons.org/licenses/by-sa/2.0/.

The reliability of the road network’s traffic volume and average speed largely depends on the reliability of the source data provided by Changchun municipal Engineering Design & Research Institute. We visualize the basic information of Changchun city, such as road network, vehicle distribution, bus stations, administrative districts, and the population and economic situation of each district, as shown in Fig. 1.

### Spatial aspects

We correct the drift data in our data correction. The heat map of the revised taxis and buses location data at 8 a.m. on Monday is shown in Fig. 1d,e, we can find that most of the vehicles are concentrated in the centre of the city, it is in accordance with the density of the city’s road network and bus stations shown in Fig. 1a,f.

**Figure 2:**
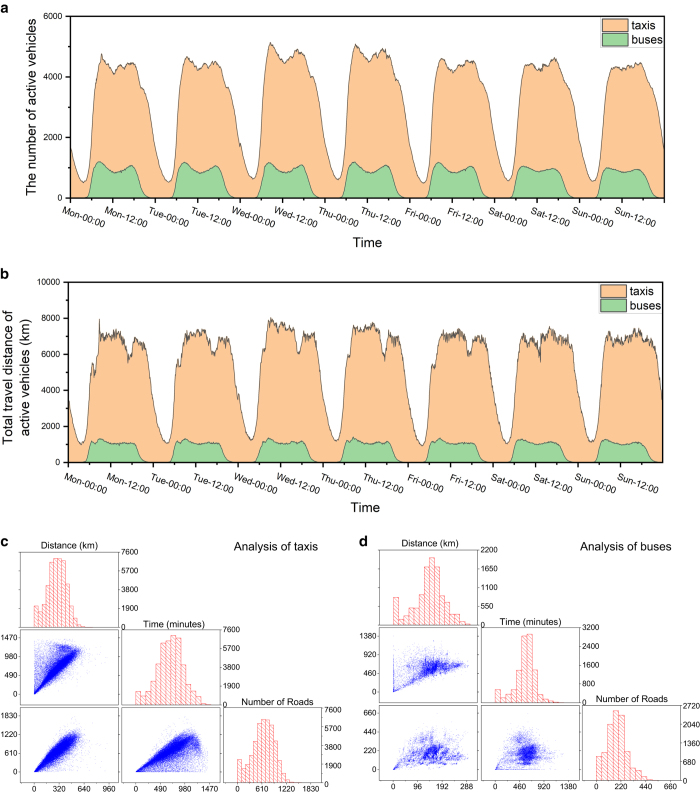
Analysis of the movement of taxis and buses. (**a**) shows the total number of active taxis and buses in every 5 minutes from Monday to Sunday. (**b**) shows the total travel distance of all active taxis and buses in every 5 minutes from Monday to Sunday. We calculate the travel distance, running time and the number of roads passed by taxis and buses, the histograms of these three features appear along the matrix diagonal, their scatter plots in the lower left corner of the matrix, as shown in (**c**) and (**d**).

**Figure 3:**
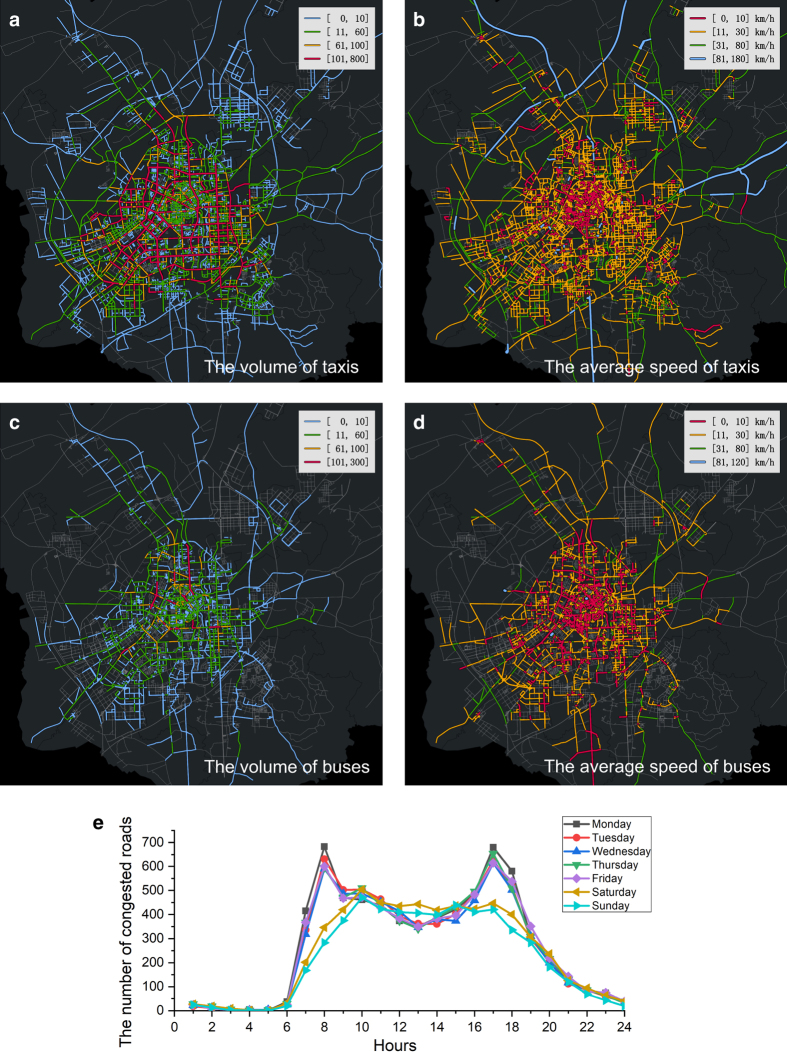
Two-layer UTN. We chose Monday morning from 8:00 am to 9:00 am as an example to show the traffic network. (**a**) shows the taxis volume network. (**b**) shows the taxi speed network. (**c**) shows the buses volume network. (**d**) shows the bus speed network. According to the distribution of volume and average speed in the road network (shown in Fig. 4), we divide traffic volume and average speed into four levels (details in spatial aspects section) and represented in different colors. (**e**) shows the number of roads with low speed and high traffic volume per hour each day. It can be found that the peak hours in the morning and evening are significantly higher than those on weekends. The spatial map was created using OpenStreetMap online platform (http://www.openstreetmap.org/) (© OpenStreetMap contributors) under the license of CC BY-SA (http://www.openstreetmap.org/copyright). More details of the licence can be found in http://creativecommons.org/licenses/by-sa/2.0/.

Then, we verify the consistency of vehicles trajectory data by the total distance of all vehicles and the number of active vehicles every five minutes, as shown in Fig. 2. We match the coordinates of vehicles to the road network, calculate the traffic volume and average speed of all roads, and build a two-layer UTN, as shown in Fig. 3.

**Figure 4:**
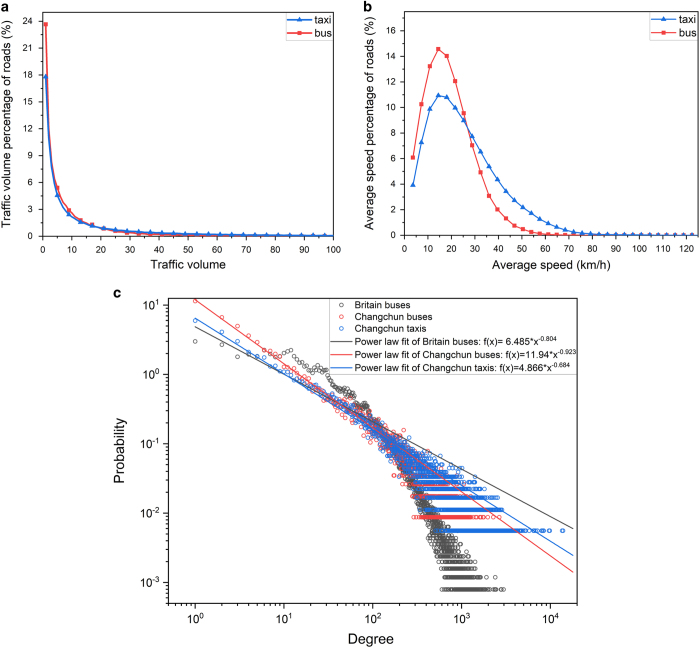
The distribution of traffic volume and average speed. We use Monday as a case study to show the distribution of traffic volume and average speed in the road network. (**a**) shows the distribution of traffic volume in the road network, the X-axis represents the traffic volume, the Y-axis represents the percentage of roads which volume equal x. (**b**) shows the distribution of average speed in the road network, the X-axis represents the average speed (km/h), the Y-axis represents the percentage of roads which average speed equal x. (**c**) shows the degree distribution of the bus-layer in Britain and the two-layer UTN in Changchun. In reference^22^, the edge weights are defined as either the minimal travel time or Euclidean distance. To make consistence with this study in Changchun, we take the bus stations as nodes and the bus lines as edges weighted by the traffic volume in bus-layer. We fit all layers by using the power-law distribution and validated by the chi-square goodness-of-fit test. We denote the degree as *x* associated with the corresponding probability as *f*(*x*). In Changchun, the bus-layer follows *f*(*x*)=11.94 ∗ *x*^−0.923^ with *r*^2^ as 0.99. The taxi-layer follows *f*(*x*)=6.485 ∗ *x*^−0.804^ with *r*^2^ as 0.97. In contrast, the bus-layer in Britain follows *f*(*x*)=4.866 ∗ *x*^−0.684^, with lower *r*^2^ as 0.73.

According to the distribution of traffic volume and average speed of in the road network (as shown in Fig. 4), we divide traffic volume into four levels: low (0 < volume ≤ 10), normal (10 < volume ≤ 60), slightly higher (60 < volume ≤ 100) and high (volume > 100). Similarly, we divide traffic speed into four levels: low (speed ≤ 10 km/h), slightly lower (10 km/h < speed ≤ 30 km/h), normal (30 km/h < speed ≤ 80 km/h) and high (speed > 80 km/h).

We can find that most of the roads with high traffic volume concentrate on several main roads in the city centre, as shown in Fig. 3a,c; most of the roads with high speed distribute in the surrounding area of the city, and most of the roads with low speed distribute in the centre area of the city, as shown in Fig. 3b,d.

### Temporal aspects

In our procedure, we correct some errors and impute the missing data and verify the consistency of the vehicles trajectory data as shown in Fig. 2. We can find some temporal characteristics in the two-layer UTN. There are two peaks, morning and evening, in total travel distance of all vehicles and the number of active vehicles (we call “activity” later) in each day, the morning peak is around 8:00 and the evening peak is around 17:00.

Moreover, the activity of the morning peak is slightly higher than that of the evening peak. The activity of taxis reaches the lowest level at 4:00, while the buses active from 5:00 to 21:00 every day. In particular, the weekend activity was lower than the working days; the total distance of taxis has a low peak in the afternoon of the working days, but not in the weekend; the total distance of buses has two peaks in the morning of workdays but only one peak in the weekend.

In addition, we count the number of roads with low speed and high traffic volume per hour in each day. We can find that the peak hours in the morning and evening are significantly higher than those on weekends, as shown in Fig. 3e.

### Multilayer aspects

We further analyse and verify the multi-layer characteristics of two-layer UTN. In Fig. 1 d,e, we can find that the distribution range of taxis is obviously larger than buses. In Fig. 2, the activity peak periods of taxis and buses are generally the same in each day. In Fig. 3, the number of roads with high volume and high speed in the taxi-layer is obviously higher than that in the bus-layer. In Fig. 4 a,b, we can find that the percentage of low volume and average speed of bus-layer is obviously higher than that in the taxi-layer.

We additionally analyse the degree distribution of volume in each layer and compare with the bus-layer in Britain^22^ as shown in Fig. 4 c. Note that, in reference^22^, the edge weights are defined as either the minimal travel time or Euclidean distance. To make consistence with this study in Changchun, according to the timetable of bus-layer in Britain (the dataset of reference^22^), we take the bus stations as nodes and the bus lines as edges weighted by the traffic volume in bus-layer. The degree of a node is defined as the traffic volume through the node in one day. Then we fit all layers by using the power-law distribution and validated by the chi-square goodness-of-fit test.

## Additional information

**How to cite this article**: Huang, Q. *et al*. The temporal geographically-explicit network of public transport in Changchun City, Northeast China. *Sci. Data*. 6:190026 https://doi.org/10.1038/sdata.2019.26 (2019).

**Publisher’s note**: Springer Nature remains neutral with regard to jurisdictional claims in published maps and institutional affiliations.

## Supplementary Material
